# Enabling translational geroscience by broadening the scope of geriatric care

**DOI:** 10.1111/acel.14034

**Published:** 2023-12-01

**Authors:** Luigi Ferrucci, David M. Wilson, Stefano Donega, Monty Montano

**Affiliations:** ^1^ Intramural Research Program of the National Institute on Aging, NIH Baltimore Maryland USA; ^2^ Biomedical Research Institute, Faculty of Medicine and Life Sciences Hasselt University Diepenbeek Belgium; ^3^ Department of Medicine Harvard Medical School Boston Massachusetts USA

**Keywords:** aging, geriatrics, geroscience, healthy longevity

## Abstract

Geroscience poses that core biological mechanisms of aging contribute to chronic diseases and disabilities in late life and that health span and longevity can be modulated by pharmacological and behavioral interventions. Despite strong evidence from studies in model organisms and great potentials for translation, most geriatricians remain skeptical that geroscience will help them in the day‐by‐day battle with the consequences of aging in their patients. We believe that a closer collaboration between gerontologists and geriatricians is the key to overcome this impasse. There is evidence that trajectories of health with aging are rooted in intrinsic and extrinsic exposures that occur early in life and affect the pace of molecular and cellular damage accumulation with aging, also referred to as the “pace” of biological aging. Tools that measure the pace of aging currently allow for the identification of individuals experiencing accelerated aging and at higher risk of multimorbidity and disability. What we term “Translational Geroscience”, i.e., the merger of fundamental and translational science with clinical practice, is thus poised to extend the action of geriatric care to a life course perspective. By targeting core mechanisms of aging, gerotherapeutics should be effective in treating patients with multimorbidity and disability, phenotypes that are all too common among geriatric patients nowadays. We call for initiatives that enhance the flow of ideas between gerontologists and geriatricians to facilitate the growth of translational geroscience. This approach can widen the scope of geriatric care, including a new role for geroscience in the promotion and operationalization of healthy longevity.

## Graphical abstract

Translational Geroscience poses that slowing down the underlying mechanisms of aging will result in prevention of most diseases and disabilities that affect older persons. To facilitate this transformation will require a closer collaboration and wider discussion between gerontologists who study aging in research laboratories (geroscience) and geriatricians who maintain life course records and care for older patients with complex multimorbidity and disability (geriatric care). Figure generated using Office suite and Biorender.

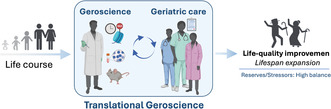

**Abbreviations**
ICD‐10: International Classification of Diseases, 10th Edition
NIH: National Institutes of Health
PRO: Patient‐reported outcome
USA: United States of America
WHO: World Health Organization

Geroscience poses that core biological mechanisms of aging (i.e., hallmarks or pillars of aging) underlie many if not all chronic diseases and disabilities associated with aging. This hypothesis is supported by multiple lines of evidence, from yeast to humans, revealing that health span and longevity can be modulated by pharmacological and behavioral interventions, albeit in a heterogeneous manner across individuals (Kennedy et al., 2014). The growing evidence that aging trajectories can be modified has fueled enthusiasm within the scientific community and the public, leading to increased interest among pharmaceutical (drug) companies and the emergence of private and public organizations that focus on regenerative medicine and healthy aging. However, the quest for the “magic pill” that can stop the effects of aging is relentless, and news about another “natural extract” or compound with miraculous properties are omnipresent in the popular press. Geroscience can be and has been a scientific and cautious response to such natural desires.

Not surprisingly, the translation to the clinic of the geroscience approach has been slow and limited. Nevertheless, we are optimistic of its potential because the scientific premises of the geroscience hypothesis are rooted in rigorous studies using model organisms and humans. We propose that formalizing a bridge between Gerontology, i.e., the investigations that define how and why we age, and Geriatrics, a medical specialty that takes care of age‐related diseases and geriatric syndromes through comprehensive geriatric assessment, has the potential to bring forth unprecedented benefits to patients and the health care system (Zampino et al., 2022). This editorial is a call‐to‐action to promote an expansive “translational geroscience” approach that benefits all individuals, including not only those who are healthy and wish to maintain their healthy status, but also older patients with varying burdens of chronic morbidity and disability.

## 1 GEROSCIENCE HAS SHIFTED FOCUS TO EARLIER IN LIFE, PRIOR TO CLINICAL PRESENTATION

A key geroscience paradigm shift is the recognition that biological damage and pathological events occur throughout the entire life course, although most medical systems currently intervene only when one or more diseases become symptomatic. Translational geroscience asserts that the pace of aging can be measured at any time over one's lifespan, permitting the identification of individuals experiencing discordance between their chronological and biological age, i.e., accelerated aging, that would benefit from more life course driven diagnostics and tailored intervention(s) that slow aging and promote healthy longevity. From this perspective, geroscience‐tailored approaches have the potential to benefit the population at large, promoting healthy aging from birth, yet also provide a path, if properly calibrated, for improving the quality of life of individuals at risk for, or burdened with, multimorbidity, i.e., the co‐occurrence of disease conditions. We believe that collaborations between Gerontologists and Geriatricians will open the door to a new medicine: precision translational geroscience, applying principles of geroscience informed by clinical practice and patient histories.

## 2 ENVIRONMENTAL DRIVERS OF AGING AND DISEASE RISK

The emerging evidence suggests that lived experiences, exposures over one's life course influence genetic, behavioral and environmental determinants of health and quality of aging. For example, events in utero and over the first few years of life influence trajectories of health and functional status later in life (Kuh et al., 2003) and can be associated with biomarkers of accelerated aging. These observations indicate that early life events and exposures can affect the underlying mechanisms of aging and alter aging trajectories. Relevant exposures, broadly defined as the exposome, include geography, family characteristics, physical and psychosocial support, nutrition, education, structural discrimination and stigma, and stress from many causes, such as air pollution, environmental toxins, natural disasters, and many others. Indeed, a growing interest is the exposome‐driven mechanisms that potentially underlie health disparities associated with race or socio‐economic status. These exposures impart a life history of damage accumulation, and short‐ and long‐term stress responses, that could potentially be imprinted in the epigenome (Baccarelli et al., 2023). To date, however, the subclinical interplay of early life exposure events with reserves and resiliencies on health trajectory remains under‐studied yet may help in explaining observed heterogeneity in health outcomes. For example, a history of long‐term engagement in physical activity may promote physical reserve and resilience that reduces disability and enhances treatment response. Similarly, an immune history of exposures and responses might predict vulnerability to future risk and adverse outcomes (Ahuja et al., 2023, Marconi et al., 2022). At present, such life history information is under‐utilized when designing treatment interventions. Thus, expanding life course epidemiological efforts, including the mechanisms that maintain allostasis (i.e., a conceptual balance of stressors and reserves) from macromolecular to whole person, will enable geriatricians to better engage in health prevention care strategies that promote healthy longevity.

## 3 A GROWING ARSENAL OF TOOLS TO MEASURE THE PACE OF AGING

Intervention outcomes in gero‐trials occur over several years or decades. To address this problem, scientists have developed molecular clocks (Hagg et al., 2019) that provide real‐time measures of the pace of biological aging as proxy measure of direct clinical outcomes such as multimorbidity or disability. Setting aside whether these clocks are mechanistically informative, it has been demonstrated that they are sensitive to various exposures during the life course. Moreover, some of the tools are sensitive to interventions, prior to evidence of impairment (Moqri et al., 2023). As a key element of translational geroscience, the growing set of molecular clocks can be used to identify and quantify the impact of differing environmental exposures and risk factors for health deterioration, and separately, inform responses to geroscience‐inspired clinical trials.

## 4 TOO MUCH EMPHASIS ON PREVENTION?

While progress in geroscience can be very exciting, it is sometime argued that the focus on prevention and detection of subclinical risk may remove the necessary attention to older people with varying levels of multimorbidity and disability. Gerontologists who have been searching for the mechanisms of aging have almost by serendipity encountered resilience pathways that allow for the maintenance of the biological stability necessary for life and function. These are mechanisms (a.k.a., hallmark/pillars) of “intrinsic” prevention of adverse modifications or damage accumulation, and failure in these mechanisms leads to molecular, cellular and biological changes that cause the phenotypes of aging, including the major chronic diseases. The objective of translational geroscience will be to use available molecular tools to define the degree of one's residual resilience capacity, as well as damage accumulation, and based on this information, engineer a precise treatment strategy that will optimize one's health span. This approach is consistent with the WHO healthy aging program (Bautmans et al., 2022), and our hope is that eventually this approach will become part of the general medical culture and a component of health provider training across medical specialties (e.g., oncology, pulmonology, cardiology, chronic infectious disease biology, etc.). However, currently, geriatricians are best positioned to open this road as they deal with the consequences of aging and chronic diseases in their everyday practice.

Gerotherapeutics promises to improve the likelihood for healthy people to remain healthy as they age, but also to effectively delay and slow down the accumulation of multimorbidity. Despite the rising prevalence of multimorbid burden worldwide, the approval of new therapeutics by regulatory agencies is disease‐specific and symptom‐focused, such that “accelerated” biological aging or “multimorbidity” are not currently considered a medical indications (The PLOS Medicine Editors, 2023). As a result, ongoing clinical trials that involve gerotherapeutics are designed to address treatment effectiveness against a specific disease rather than as a intervention against unhealthy aging per se. Looking forward, the translational geroscience approach may dissolve the existing boundaries around a single disease mindset and open the focus to improving health and function in persons across a spectrum of health states, from disease‐free with future risk to prevalent multimorbidity.

## 5 RE‐CALIBRATING GEROSCIENCE TO PREVENT AND TREAT MULTIMORBIDITY AND DISABILITY

Multimorbidity can be heterogeneous with varying trajectories over the life course (Vetrano et al., 2020). Geriatricians have developed modalities of care for this population, and by focusing on function and subjective goals of care (e.g., PROs: patient defined reported outcomes), have improved the quality of life for many patients. In a society that celebrates the heroics of “saving a life”, exemplified by television programs such as “ER” or “House”, or that marvels at multimillionaires leveraging geroscience to maintain their optimal health (https://www.newyorker.com/magazine/2017/04/03/silicon‐valleys‐quest‐to‐live‐forever), activities that occur in a context of growing health inequity (Zaninotto et al., 2020), there is proportionately less attention given to those whose health is fading over time. Today, patients with multimorbidity and/or disability account for an increasing bulk of the pathology and disease burden in our population.

Can translational geroscience help improve the care of geriatric patients and bring more doctors to the geriatric specialty? Few people think about engaging a geriatrician until they happen to have a close family member or friend experiencing multimorbidity or a geriatric syndrome (e.g., frailty, dementia). However, as pointed out by a recent editorial in JAMA by Dr. Gurwitz, despite the growing need, geriatrics as a profession is paradoxically becoming less popular in the United States (Gurwitz, 2023). Moreover, geriatricians are among the least paid specialty in the entire health care system, dampening enthusiasm for pursuing this profession. Geriatricians' unique expertise will be essential to the design and real‐world implementation of gerotherapeutics and to move new discoveries from the proverbial bench to bedside, ultimately guiding improved routine health care. The new emphasis on the biological mechanisms of aging may also spark a new interest for geriatric medicine throughout one's life course and offer an opportunity for professional and financial growth through an expanded relevance across the human condition. At present, many geriatricians are skeptical that the new geroscience can help them in their everyday practice, largely because of the lack of evidence for translation from bench to bedside. We argue that we are still in the early stage of the translational geroscience transformation, but the window of opportunity opened by geroscience has clear potential to lead to profound therapeutic advances, with two major factors supporting our optimism. First, gerotherapeutics aim to empower the cellular, organismal and whole person mechanisms that maintain an allostatic equilibrium (defined as a balance between the accumulation of lived biological and psychosocial stressors countered by resiliencies and reserve). Second, the development of tools that measure the biology of aging provide decision‐enabling tools to measure a person's “biological aging profile”. Information regarding the status of an individual's resilience capacities and biological age could inform therapeutic approaches. For example, the accumulation of senescent cells, an age‐related biomarker, within a specific organ may serve as a predictor of inflammaging and adverse response to chemotherapy (Demaria et al., 2017). In spite of the complexity implicit in the design and implementation of clinical trials with older persons, the translation of some geroscience discoveries is occurring, with some clinical trials on senolytic candidates already in the field (Rolland et al., 2023; Wyles et al., 2022).

## 6 AN OPPORTUNITY TO INTEGRATE GERIATRICS INTO GEROSCIENCE‐INSPIRED THERAPEUTIC STRATEGIES ACROSS THE LIFE COURSE

For translational geroscience to advance, we will need to open communication and stimulate collaboration between gerontologists and geriatricians. We need a serious commitment on both sides to join the “discussion at the table” to capitalize on this emerging opportunity, as breeching the gap between geroscientists and clinicians will require reciprocal cooperation. For example, it happens too often that fundamental, basic scientists that discuss geroscience in scientific meetings do so to an audience of their peers, with little participation from clinicians. Moreover, to accelerate patient‐driven progress, we believe that geriatricians who are fully aware of the problems of older patients could communicate these complex problems and broader needs to gerontologists. Luckily, we do not have to start from ground zero, as initiatives aimed at encouraging interactions and shared discussion have already begun. As a few key examples, we note the following: (i) A discussion on the value of geroscience is ongoing in the Association of Directors of Geriatric Academic Programs, and such discussion has broadened to the American Geriatric Society at large. (ii) The third and fourth Geroscience Summits organized by the National Institute on Aging brought together researchers and health professionals from various disciplines to discuss how to advance the translational geroscience agenda. However, we need additional and broader efforts of this sort, as well as new creative approaches. For example, scientists who study aging may benefit from direct contact with geriatricians and geriatric patients. Similarly, building into the geriatric curriculum an opportunity for geriatricians to work in research laboratories that focus on translational geroscience issues may help in breaking down skepticism.

One example of how a translational geroscience approach might be aligned to benefit patients is in the management of sarcopenia, a heterogeneous condition that involves known biological drivers of aging. There has been an enormous effort over the last several years to agree on a diagnosis of sarcopenia, combining information on strength, body mass, walking speed and other parameters. However, there remains little agreement on diagnostic criteria nor an effective treatment, despite the successful introduction of an ICD‐10 code. An alternative approach would be to recognize sarcopenia as an aging syndrome caused by damage accumulation across multiple physiological domains, including the central and peripheral nervous, cardiorespiratory, vascular and osteoarticular systems. In this scenario, instead of focusing on muscle characteristics, interventions would focus on the multiple health trajectories leading to sarcopenia that variably involve pathways related to the aging hallmarks. The variability in disease complexity of course lies on top of many other age and life history dependent factors. We posit that just a simple history of the level of physical activity in the different epochs of life may be extremely informative in understanding the antecedents to risk for sarcopenia. Formal recordings of specific life course events or exposures using emerging tools, coupled with an understanding of how these experiences affect sarcopenia through the lens of translational geroscience, would likely be transformative in the way geriatricians assess and tackle this condition. Envisioned gerotherapeutics would, in principle, target multiple biological systems to improve muscle function in many individuals, although potentially through distinct mechanisms in different persons.

## 7 A NEW PARTNERSHIP ON THE HORIZON

We started this discussion with an optimistic perspective; namely, that translational geroscience, through integration of a targeted medical history with molecular aging diagnostics, can turn a page in medicine and expand the reach of geriatrics. A question posed by Richard Miller in 1997, “When will the biology of aging become useful?”, is more relevant now than ever (Miller, 1997). We acknowledge that today there are only limited examples of treatments coming from geroscience in clinical practice, as we wait for the results of the many clinical trials that are currently ongoing (Chaib et al., 2022; Raffaele & Vinciguerra, 2022). Nevertheless, our hope, with the expectation that gerotherapeutics will become more common place as we move forward, is that we align efforts to better understand aging trajectories and better enable geroscience‐informed geriatrics to promote healthy aging. Indeed, a growing number of older persons burdened by frailty and other aging syndromes are waiting for us to recognize and address their need. There is no time or energy to waste, and we hope geroscientists and geriatricians will join forces to champion a translational geroscience transformation in healthcare.

## AUTHOR CONTRIBUTIONS

Luigi Ferrucci and Monty Montano developed the idea and wrote the initial draft. David M Wilson III and Stefano Donega revised the text and Stefano Donega drafted the Figure.

## CONFLICT OF INTEREST STATEMENT

None of the authors have conflicts of interest relative to this article.

## Data Availability

I confirm that my article contains a Data Availability Statement even if no new data was generated (list of sample statements) unless my article type does not require one.

## References

[acel14034-bib-0001] Ahuja, S. K. , Manoharan, M. S. , Lee, G. C. , McKinnon, L. R. , Meunier, J. A. , Steri, M. , Harper, N. , Fiorillo, E. , Smith, A. M. , Restrepo, M. I. , Branum, A. P. , Bottomley, M. J. , Orrù, V. , Jimenez, F. , Carrillo, A. , Pandranki, L. , Winter, C. A. , Winter, L. A. , Gaitan, A. A. , … He, W. (2023). Immune resilience despite inflammatory stress promotes longevity and favorable health outcomes including resistance to infection. Nature Communications, 14(1), 3286. 10.1038/s41467-023-38238-6 PMC10264401 37311745

[acel14034-bib-0002] Baccarelli, A. , Dolinoy, D. C. , & Walker, C. L. (2023). A precision environmental health approach to prevention of human disease. Nature Communications, 14(1), 2449. 10.1038/s41467-023-37626-2 PMC10147599 37117186

[acel14034-bib-0003] Bautmans, I. , Knoop, V. , Amuthavalli Thiyagarajan, J. , Maier, A. B. , Beard, J. R. , Freiberger, E. , Belsky, D. , Aubertin‐Leheudre, M. , Mikton, C. , Cesari, M. , Sumi, Y. , Diaz, T. , Banerjee, A. , & WHO Working Group on Vitality Capacity . (2022). WHO working definition of vitality capacity for healthy longevity monitoring. The Lancet Healthy Longevity, 3(11), e789–e796. 36356628 10.1016/S2666-7568(22)00200-8 PMC9640935

[acel14034-bib-0004] Chaib, S. , Tchkonia, T. , & Kirkland, J. L. (2022). Cellular senescence and senolytics: The path to the clinic. Nature Medicine, 28(8), 1556–1568. 10.1038/s41591-022-01923-y PMC9599677 35953721

[acel14034-bib-0005] Demaria, M. , O'Leary, M. N. , Chang, J. , Shao, L. , Liu, S. , Alimirah, F. , Koenig, K. , le, C. , Mitin, N. , Deal, A. M. , Alston, S. , Academia, E. C. , Kilmarx, S. , Valdovinos, A. , Wang, B. , de Bruin, A. , Kennedy, B. K. , Melov, S. , Zhou, D. , … Campisi, J. (2017). Cellular senescence promotes adverse effects of chemotherapy and cancer relapse. Cancer Discovery, 7(2), 165–176. 27979832 10.1158/2159-8290.CD-16-0241 PMC5296251

[acel14034-bib-0006] Gurwitz, J. H. (2023). The paradoxical decline of geriatric medicine as a profession. JAMA, 330(8), 693–694. 37540519 10.1001/jama.2023.11110

[acel14034-bib-0007] Hagg, S. , Belsky, D. W. , & Cohen, A. A. (2019). Developments in molecular epidemiology of aging. Emerging Topics in Life Sciences, 3(4), 411–421. 33523205 10.1042/ETLS20180173 PMC7289014

[acel14034-bib-0008] Kennedy, B. K. , Berger, S. L. , Brunet, A. , Campisi, J. , Cuervo, A. M. , Epel, E. S. , Franceschi, C. , Lithgow, G. J. , Morimoto, R. I. , Pessin, J. E. , Rando, T. A. , Richardson, A. , Schadt, E. E. , Wyss‐Coray, T. , & Sierra, F. (2014). Geroscience: Linking aging to chronic disease. Cell, 159(4), 709–713. 25417146 10.1016/j.cell.2014.10.039 PMC4852871

[acel14034-bib-0009] Kuh, D. , Ben‐Shlomo, Y. , Lynch, J. , Hallqvist, J. , & Power, C. (2003). Life course epidemiology. Journal of Epidemiology and Community Health, 57(10), 778–783. 14573579 10.1136/jech.57.10.778 PMC1732305

[acel14034-bib-0010] Marconi, V. C. , Krishnan, V. , Ely, E. W. , & Montano, M. (2022). Immune health grades: Finding resilience in the COVID‐19 pandemic and beyond. The Journal of Allergy and Clinical Immunology, 149(2), 565–568. 34740606 10.1016/j.jaci.2021.10.025 PMC8560746

[acel14034-bib-0011] Miller, R. A. (1997). When will the biology of aging become useful? Future landmarks in biomedical gerontology. Journal of the American Geriatrics Society, 45(10), 1258–1267. 9329491 10.1111/j.1532-5415.1997.tb03781.x

[acel14034-bib-0012] Moqri, M. , Herzog, C. , Poganik, J. R. , Justice, J. , Belsky, D. W. , Higgins‐Chen, A. , Moskalev, A. , Fuellen, G. , Cohen, A. A. , Bautmans, I. , Widschwendter, M. , Ding, J. , Fleming, A. , Mannick, J. , Han, J. D. J. , Zhavoronkov, A. , Barzilai, N. , Kaeberlein, M. , Cummings, S. , … Gladyshev, V. N. (2023). Biomarkers of aging for the identification and evaluation of longevity interventions. Cell, 186(18), 3758–3775. 37657418 10.1016/j.cell.2023.08.003 PMC11088934

[acel14034-bib-0013] Raffaele, M. , & Vinciguerra, M. (2022). The costs and benefits of senotherapeutics for human health. The Lancet Healthy Longevity, 3(1), e67–e77. 36098323 10.1016/S2666-7568(21)00300-7

[acel14034-bib-0014] Rolland, Y. , Sierra, F. , Ferrucci, L. , Barzilai, N. , de Cabo, R. , Mannick, J. , Oliva, A. , Evans, W. , Angioni, D. , de Souto Barreto, P. , Raffin, J. , Vellas, B. , Kirkland, J. L. , the G.C.T‐TF group , Andrieu, S. , Bacqueville, D. , Bischoff‐Ferrari, H. , Blivet, G. , Cash, T. , … Zhavoronkov, A. (2023). Challenges in developing Geroscience trials. Nature Communications, 14(1), 5038. 10.1038/s41467-023-39786-7 PMC10439920 37598227

[acel14034-bib-0015] The PLOS Medicine Editors . (2023). Multimorbidity: Addressing the next global pandemic. PLoS Medicine, 20(4), e1004229. 37014909 10.1371/journal.pmed.1004229 PMC10072481

[acel14034-bib-0016] Vetrano, D. L. , Roso‐Llorach, A. , Fernandez, S. , Guisado‐Clavero, M. , Violán, C. , Onder, G. , Fratiglioni, L. , Calderón‐Larrañaga, A. , & Marengoni, A. (2020). Twelve‐year clinical trajectories of multimorbidity in a population of older adults. Nature Communications, 11(1), 3223. 10.1038/s41467-020-16780-x PMC7320143 32591506

[acel14034-bib-0017] Wyles, S. P. , Tchkonia, T. , & Kirkland, J. L. (2022). Targeting cellular senescence for age‐related diseases: Path to clinical translation. Plastic and Reconstructive Surgery, 150, 20S–26S. 10.1097/PRS.0000000000009669 PMC9529239 36170432

[acel14034-bib-0018] Zampino, M. , Polidori, M. C. , Ferrucci, L. , O'Neill, D. , Pilotto, A. , Gogol, M. , & Rubenstein, L. (2022). Biomarkers of aging in real life: Three questions on aging and the comprehensive geriatric assessment. Geroscience, 44(6), 2611–2622. 35796977 10.1007/s11357-022-00613-4 PMC9261220

[acel14034-bib-0019] Zaninotto, P. , Batty, G. D. , Stenholm, S. , Kawachi, I. , Hyde, M. , Goldberg, M. , Westerlund, H. , Vahtera, J. , & Head, J. (2020). Socioeconomic inequalities in disability‐free life expectancy in older people from England and the United States: A cross‐national population‐based study. The Journals of Gerontology: Series A, Biological Sciences and Medical Sciences, 75(5), 906–913. 31940032 10.1093/gerona/glz266 PMC7164527

